# The Anticancer Application of Delivery Systems for Honokiol and Magnolol

**DOI:** 10.3390/cancers16122257

**Published:** 2024-06-18

**Authors:** Katarzyna Dominiak, Aleksandra Gostyńska, Michał Szulc, Maciej Stawny

**Affiliations:** 1 Department of Pharmaceutical Chemistry, Poznan University of Medical Sciences, Rokietnicka 3, 60-806 Poznań, Poland; agostynska@ump.edu.pl; 2 Department of Pharmacology, Poznan University of Medical Sciences, Rokietnicka 3, 60-806 Poznań, Poland; mszulc@ump.edu.pl

**Keywords:** honokiol, nanoparticles, drug delivery system, cancer, bioactive natural compounds

## Abstract

**Simple Summary:**

The rising incidence of cancer is a major threat to patients and healthcare services worldwide. It is therefore important to search for novel therapies and new drugs. Honokiol, found in the bark of the magnolia tree, has many properties, including anticancer activity. However, its potential use is limited due to its poor solubility in water. The present study aimed to review the available studies using modern delivery systems for honokiol and determine their anticancer efficacy in vitro and in vivo.

**Abstract:**

Cancer is a leading cause of death worldwide, and the effectiveness of treatment is consistently not at a satisfactory level. This review thoroughly examines the present knowledge and perspectives of honokiol (HON) in cancer therapeutics. The paper synthesizes critical insights into the molecular mechanisms underlying the observed anticancer effects, emphasizing both in vitro and in vivo studies. The effects of HON application, primarily in the common types of cancers, are presented. Because the therapeutic potential of HON may be limited by its physicochemical properties, appropriate delivery systems are sought to overcome this problem. This review discusses the effect of different nanotechnology-based delivery systems on the efficiency of HON. The data presented show that HON exhibits anticancer effects and can be successfully administered to the site of action. Honokiol exerts its anticancer activity through several mechanisms. Moreover, some authors used the combinations of classical anticancer drugs with HON. Such an approach is very interesting and worth further investigation. Understanding HON’s multiple molecular mechanisms would provide valuable insights into how HON might be developed as an effective therapeutic. Therefore, further research is needed to explore its specific applications and optimize its efficacy in diverse cancer types.

## 1. Introduction

**Figure 1:**
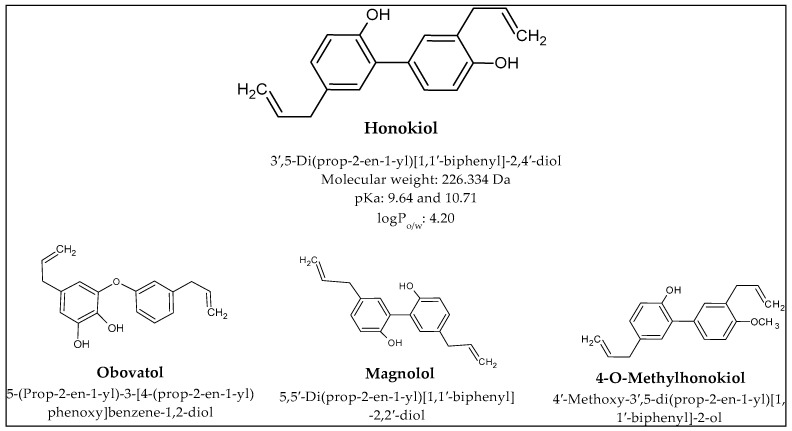
Structure and properties of magnolia-derived bioactive compounds.

Traditional Asian medicine is based on plant-origin compounds, among which magnolia-derived substances can be found. Two species are mainly used to isolate substances from magnolia—*Magnolia officinalis* and *Magnolia obovate* [1]. Magnolia contains approximately 255 compounds from different chemical groups such as flavonoids, coumarins, alkaloids, terpenoids, neolignans, and phenylpropanoids. However, it is the neolignans that are responsible for the main health benefits, which include honokiol (HON), magnolol, 4-O-methylhonokiol, and obovatol (Figure 1).

The content of individual compounds depends on the species of magnolia [2]. The extraction of neolignans is possible from the bark of different plant parts, such as the trunk, root, or branches. The content of each compound in a given part of the plant is influenced by its area of origin, altitude, magnolia species, and parts of the plant from which the bark is obtained. The content of compounds can vary up to 10 times, taking into account all the factors mentioned above. The bark, which contains the main compounds, can be obtained from the root, branches, or trunk. Nevertheless, it has been shown that the bark from the magnolia roots contains the highest amount of HON and magnolol, ranging from 87 to 96 mg/g. However, HON has attracted more and more attention due to its pleiotropic effect and therapeutic potential. Regarding structure, HON belongs to a class of neolignan biphenols and is a positional isomer of magnolol. LD50 (lethal dose 50) is the dose at which half of the organisms die after administration of the substance. According to the National Research Institute of Chinese Medicine, the LD50 for magnolia bark extract is >50 g/kg b.w (body weight). However, the intraperitoneal (i.p.) dose is lower at 8.5 g/kg b.w. [1]. One study investigated the acute and chronic toxicity of honokiol microemulsions. The authors found that the LD50 for lignan was 50.5 mg/kg body weight in mice [3]. In the solid state, HON is a powder with a melting point of 87 °C. It is a hydrophobic compound, so its solubility in water is limited. Nevertheless, due to the presence of hydroxyl groups in the molecule structure of HON, the pH of the solution affects its solubility, leading to its better solubility in acidic pH [4]. Evidence suggests that HON possesses anti-inflammatory, antioxidant, neuroprotective, anticancer, and cardioprotective properties [5]. Therefore, it has high therapeutic potential.

In addition, HON can easily cross the blood–brain and blood–cerebrospinal fluid barriers, further expanding its possible medicinal application [6]. HON is metabolized in the liver and undergoes glucuronidation, sulphation, and oxidation. Several metabolites are formed as a result, but the main ones include monoglucuronide and monosulfate [7].

Because of the poor water solubility of HON, its therapeutic potential is limited. Therefore, to overcome this problem, the development of appropriate delivery systems is needed. Modern delivery systems not only allow the utilization of poorly water-soluble compounds but also may reduce side effects, improve transport to the target site, and extend the duration of the compound’s action through controlled release. These properties make such systems an excellent solution for effective drug delivery, especially in cancer treatment. Several nanosystems are available, including micelles, liposomes, polymeric nanoparticles, mesoporous silica nanoparticles, and nanotubes. Their use in therapeutics can help overcome various limitations of anticancer therapy, enabling more precise cancer treatment, more convenient monitoring of therapeutic agents, and even overcoming anticancer drug resistance. Moreover, most nanotechnology-based drug delivery systems allow for many properly designed and reproducible therapeutic formulations, which gives a good prognosis for the possibility of scaled-up production [8].

This review aimed to summarize the currently available literature, in which the authors evaluated the efficacy of HON delivery systems in different cancers. The performance evaluation of these systems included effects on cancer cells in vitro and in vivo.

## 2. Breast Cancer

Breast cancer (BC) is one of the most common cancers in the female population. According to the European Cancer Information System (ECIS) data, 1/11 women will develop BC under 74 years of age in Europe. In men, this risk is much lower, reaching only 1/909. Further ECIS statistics based on the observations in 2022 show a BC incidence of 190/100,000, of whom 45/100,000 died [9]. One of the problems encountered in cancer treatment is the resistance of cells to selected therapies. For this reason, on the one hand, it is crucial to search for new substances that exhibit anticancer activity, as well as to develop nanosystems that will enable targeted therapies [10]. HON has been widely evaluated against BC cells in in vitro and in vivo studies. The activity of a variety of delivery systems such as liposomes [11,12,13,14], micelles [15,16,17,18], mixed micelles [19], nanocapsules [20], nanoparticles [21,22,23,24,25,26], or nanosuspension-loaded thermosensitive hydrogels [27] loaded with this compound were tested. The main findings concerning the effect of such systems in different BC models are presented in Table 1.

As shown in Table 1, different assisted drugs with HON were used, which makes it difficult to compare the results achieved. On the other hand, different carriers were used as transporters, which may be essential in the differences in the observations. In the case of HON-loaded liposomes, anticancer effects were mainly evaluated in vitro on 4T1 cells and in vivo on BALB/c mice [11,12,13]. All developed liposomes contained additional compounds that modified their properties, leading to an increased cytotoxic effect. In one of the studies [11], the surface of the liposomes was modified by the attachment of polysialic acid. The addition of this polysaccharide resulted in increased cellular uptake of the liposomes by the endocytosis process. The IC50 values for HON-loaded liposomes without polysialic acid and HON-loaded liposomes with attached acid were 10.27 ± 0.29 μg/mL and 4.84 ± 0.18 μg/mL, respectively. This confirmed the hypothesis that polysialic acid reduces the required concentration of HON for exciting sufficient antitumor activity. Inhibitory ratios of tumor volumes were 52.40% for the prepared liposomes. At the same time, this ratio was equal to 22.56% and 33.04% for free HON and lignan liposomes without polysialic acid, respectively. Liposomes containing HON and polysialic acid prevented the formation of lung metastases in the mice studied. This study demonstrated the antitumor activity of HON and the benefits of polysialic acid attached to the surface of liposomes.

A similar study was carried out evaluating the anticancer effects of HON liposomes with hyaluronic acid. Similar to the previous experiment, the designed liposomes accumulated better in the tumor, prevented metastasis, and inhibited tumor growth more strongly [12]. Wenli Hou et al. [13] determined the enhancement of the antitumor effect of adriamycin combined with HON in liposomes. This combination allowed a decrease in the IC50 compared to adriamycin. It also strongly induced apoptosis, which was 3- and 2-fold higher than each compound alone. The prepared liposomes extended the lifespan of the mice by more than 60 days in the in vivo studies. HON and adriamycin used separately provided survival of only up to 43 days. Another study evaluated liposomes with HON and daunorubicin modified with hyaluronic acid [14]. The antitumor effect was characterized by inhibiting the formation of microvascular channels (vasculogenic mimicry, VM). The developed liposomes more efficiently inhibited VM formation and reduced the expression of VM indicators such as focal adhesion kinase (FAK), matrix metalloproteinase-2 (MMP-2), matrix metalloproteinase-9 (MMP-9), and ephrin type-A receptor 2 (EphA2). The in vivo studies were performed in BALB/c mice with developed tumors from MDA-MB-435S cells. The modified liposomes had a strong accumulation in tumor cells up to 24 h after administration. The prepared drug-loaded system inhibited tumor growth better than other groups without causing systemic toxicity. The polymeric micelles loaded with the combination of HON and paclitaxel were designed to evaluate their efficacy on BC cells [15,16]. Studies were performed in vitro on 4T1 and HEK293 cells [15] and MCF-7/ADR, MDA-MB-231 cell lines [16]. The efficacy of the designed micelles was also evaluated in the in vivo studies in BALB/c mice. It was determined that the IC50 value (against MCF-7/ADR cells) was more than 3-fold lower when the mass ratio of paclitaxel to HON was 1:1. Tests on mice injected intravenously with MDA-MB-231-luc-GFP cells allowed for the observation of tumor metastasis. A bioluminescence imaging system and images of the excised lungs confirmed that micelles with HON and paclitaxel prevented metastasis better than using the anticancer drug alone [16]. Micelles with HON and paclitaxel increased the number of apoptotic cells to 34.02 ± 0.05%. The in vivo studies allowed the observation of tumor growth inhibition. The tumor volume was 557.64 ± 243.81 mm^3^ for the designed micelles, while for micelles with HON or paclitaxel, it was only 1463.77 ± 148.74 mm^3^ and 932.70 ± 256.43 mm^3^, respectively [15]. 

Yang Zou et al. [17] evaluated polymeric micelles of HON with doxorubicin on MDA-MB-231 cells. The best in vitro results had micelles containing lignan and doxorubicin at the mass ratios of 3:1 and 5:1. Therefore, they were included in the in vivo studies. According to the hypothesis, the process of cancer cell invasion was most strongly inhibited by the prepared micelles. A bioluminescence signal determined the limitation of lung metastasis formation. Jiahui Sun et al. [19] developed methoxy poly(ethylene glycol)-poly(lactide) (mPEG-PLA)/D-α-tocopherol polyethylene glycol 1000 succinate (TPGS)-based mixed micelles to encapsulate HON and celecoxib. As in previous examples, the effect of the micelle solution on BC cells was evaluated in vitro and in vivo in mouse models. A cytotoxic effect was confirmed but did not involve healthy tissues. Micelles reduced the expression of biomarkers that include forkhead box P3 (Foxp3), cluster of differentiation 4 (CD4), granulocyte differentiation antigen-1 (Gr-1), cluster of differentiation 11b (CD11b), cluster of differentiation 31 (CD31), antigen Ki-67 (Ki67), forkhead box M1 (FoxM1), and vascular endothelial growth factor (VEGF) [19]. In another study, nanocapsules were prepared from PEGylated poly(lactic-co-glycolic acid) (PLGA) [20]. The nanoprecipitation method was used to obtain them and allowed the preparation of nanocapsules with suitable physicochemical properties. The nanocapsules with the lowest particle sizes and zeta potential values (but containing the highest PEG content) had the highest cellular uptake of 71.42 ± 6.94%. The HON-loaded nanocapsules had a cytotoxic effect that persisted for the next three days of the study. The IC50 value on MCF-7 cells was 20 ± 2.3 μM after 24 h, while the IC50 of free HON was 52.63 ± 5.4 μM. The in vivo studies were performed on mice with a solid Ehrlich carcinoma breast cancer model. Tumor growth inhibition, tumor weight, effects on signaling pathways, and systemic toxicity were assessed during the experiments. In the group treated with free HON, the tumor size was 906.75 ± 105.32 mm^3^, while with nanocapsules, it was 266.89 ± 115.69 mm^3^. The percentage inhibition of tumor growth was 35% and 80.85%, for free HON and its nanocapsules, respectively. ELISA tests confirmed the anti-angiogenic and apoptotic activities of the nanocapsules through the regulation of signaling pathways VEGF-1 and caspase-3 and b-cell lymphoma 2 (Bcl-2). After systemic administration, the prepared system had no toxic effects on the liver and kidneys. Zein nanoparticles modified with polysialic acid have also been developed to encapsulate HON. The IC50 value for the surface-modified nanoparticles was 4.37 μg/mL, while the value without polysialic acid was 7.74 μg/mL. The tumor was significantly reduced using nanoparticles with attached acid. The improved antitumor effect of the designed system was also confirmed in the in vivo experiments [21]. In another study, HON nanoparticles were developed with zein and hyaluronic acid. The system had a better anticancer effect compared to the pure compound [22]. This lignan was also encapsulated with polidopamine and folic acid. The IC50 value was almost four times lower compared to the control group. At the same time, the nanoparticles did not cause systemic toxicity [23]. Another way to increase HON’s solubility was to develop a lignan nanosuspension and load it into a thermosensitive hydrogel. The system was then evaluated in BC trials. It was used in combination with intravenous paclitaxel. Both compounds yielded very good results in the tests. However, the best results were achieved with free paclitaxel with nanosuspension HON in hydrogel (40 mg/kg). The tumor inhibitory rate was 72.51%, while the number of apoptotic cells increased to more than 65%. Moreover, HON increased the concentration of paclitaxel in the tumor. A combined therapy with paclitaxel and a novel HON delivery system gave excellent results in vitro and in vivo [27].

**Figure 2:**
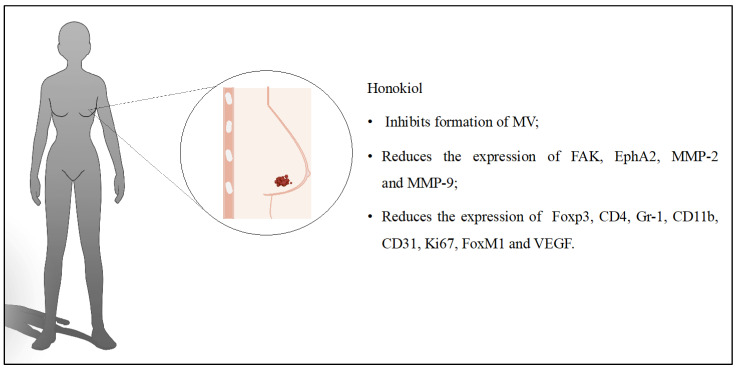
Key findings related to breast cancer.

Several subtypes of BC are currently distinguished. One of these is triple-negative breast cancer (TNBC), which accounts for up to 15% of all BC cases. The absence of estrogen and progesterone receptors and the poor expression of the human epidermal growth factor receptor 2 characterize TNBC. In TNBC, tumor cells divide rapidly and often metastasize [28]. Current treatments are not fully satisfactory. However, there is hope that targeted therapies could improve the prognosis for patients. The effectiveness of HON micelles in the treatment of TNBC was also assessed. The in vitro studies confirmed a cytotoxic effect on BC cells and increased bioavailability. Tests in mice proved that the developed micelles reduced angiogenesis, tumor volumes, and weights compared to the group receiving free HON [18]. The discussed influence of HON on BC is presented in Figure 2.

## 3. Glioblastoma

Gliomas, including glioblastoma multiforme (GBM), are among the most malignant central nervous system tumors. Despite advances in treatment modalities, it remains mainly incurable, with one of the highest mortality rates. Hence, the search for new treatment strategies has become very important [29]. The treatment of gliomas, including glioblastoma multiforme, is hampered by a suppressive tumor-immune microenvironment (TIME). Attempts to modify treatment have focused on finding new drugs and designing delivery systems that more easily pass through the blood–brain barrier. The summary of HON formulations developed for glioma treatment is presented in Table 2.

Zening Zheng et al. [30] developed liposomes loaded with HON and disulfiram/copper complex. Lignan was selected for its potential to inhibit the mammalian target of rapamycin (mTOR), which directly affects TIME. In addition, a ^D^CDX peptide with a strong affinity for nicotinic acetylcholine receptors was included. These receptors are highly expressed in the endothelial cells of brain tumor capillaries. The developed liposomes were characterized by a spherical shape with an average size of 122.5 nm. Encapsulation efficiency was above 85%. HON, not combined with disulfiram/copper, had limited cytotoxicity, as indicated by the IC50 values of 20.1 (U87 cells) and 13.3 μg/mL (C6 cells). The lignan and disulfiram/copper complex-loaded liposomes showed excellent antitumor efficacy (IC50 = 0.16 μg/mL for U87 cells). The designed system inhibited protein kinase B (Akt)/mTOR activity, contributing to the reduction in tumor growth. In addition, the measurement of autophagic proteins such as microtubule-associated protein 1 light chain 3-II (LC3-II), autophagy-related 5 (Atg5), lysosomal-associated membrane protein 1 (LAMP1), and Beclin-1 indicated cell death after liposome treatment. During the in vivo studies, the survival time of mice was extended to 27 days. As many as 30% of them were alive after the experiment. The study results confirmed the validity of the hypothesis that developed liposomes may be effective in treating glioblastoma by affecting mTOR, autophagy, and tumor metabolism [30].

**Figure 3:**
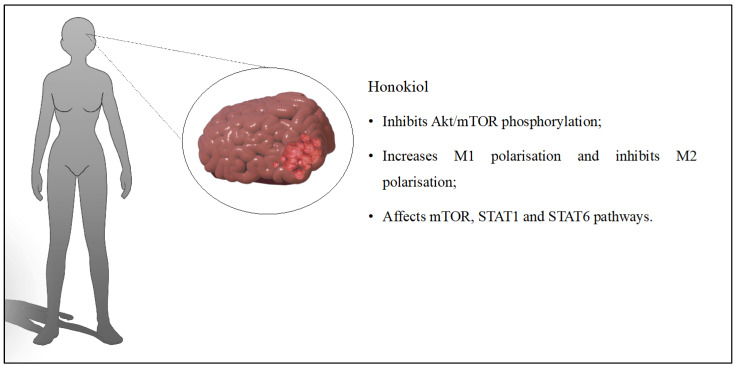
Key findings related to glioblastoma.

Glioblastoma also presents glioma-associated microglia/macrophages, which can be in two states of activation: M1 and M2. M1 macrophages cause the inhibition of tumor activity, while M2 has the opposite effect—promoting tumor activity. Therefore, the effect of liposomal HON on macrophage polarization was studied by Shenglan Li et al. [31]. It was indicated that the system, at concentrations ranging from 2.5 to 10 μM, affected the polarization of M1 macrophages, while not promoting their proliferation. HON inhibited M2 phenotype differentiation of macrophages in liposomes. By affecting the signal transducer and activator of the transcription 1 and 6 (STAT1/6) pathways, the system modified M1/M2 polarization. The in vivo studies indicated a reduction in M2 macrophage infiltration into the tumor and increased M1 macrophage accumulation. The study confirmed that the antitumor effect of liposomal HON is related to macrophage polarization [31]. In another study, the liposomal formulation of HON and dounorubicin was developed. In addition, its surface was modified with lactoferrin, whose receptors are overexpressed in glioma cells. The liposomes were characterized by a spherical shape with a size of 100 nm. The prepared system exhibited strong inhibition on C6 cells (42.88 ± 3.02%). The number of invasive cells for the empty liposomes was 153.67 ± 4.51, while for the developed system, it was 60.67 ± 5.51. The in vitro studies further confirmed the destructive effect of liposomes on the formation of VM channels (which are responsible for delivering nutrients to the tumor). The liposomes used did not show any systemic toxicity in vivo. Mouse survival time with lactoferrin-modified liposomes containing HON and daunorubicin was the longest (23–51 days) compared to control groups. This was an indication of their high potential for further research. In conclusion, the liposomes obtained promising results in the studies that were conducted, which were related to several aspects. Firstly, the liposomes had very good physicochemical properties, and the system persisted in the body for up to 24 h. Secondly, lactoferrin increased transport into tumor cells, while HON inhibited cell invasion and reduced the formation of VM channels [32]. HON was combined with doxorubicin in a similar study to investigate their effects on glioma. Micelles were designed and produced using methoxy poly(ethylene glycol)-poly(ε-caprolactone) (MPEG-PCL). In the first step, the physicochemical properties were evaluated. Then, their effects were determined in vitro on C6 cells and in vivo on mouse and zebrafish model embryos. The encapsulation efficiency was 93.4% for doxorubicin and 99.8% for lignan. To compare the effect of HON micelles with doxorubicin, micelles were additionally produced with only one substance or the other. Micelles containing doxorubicin and a combination of both compounds only had a cytotoxic effect. Nevertheless, the combination of HON and doxorubicin had greater cytotoxicity. The system increased the number of apoptotic cells compared to the other groups. Studies on transgenic zebrafish model embryos proved the inhibition of blood vessel growth and tumor shrinkage. Thus, both in vitro and in vivo studies indicated that micelles affected the apoptosis, angiogenesis, and toxicity of cancer cells [33]. Wang Ce et al. [34] had the opportunity to evaluate the safety and efficacy of HON-loaded liposomes included in the treatment plan of a patient with recurrent glioma. A 36-year-old man diagnosed with glioma was treated according to guidelines. After three months, liposomal HON was administered in a single dose of 420 mg. Side effects were not observed three days after administration. Therefore, a second phase was initiated, during which the patient received 420 mg of liposomes for five days, with two days off. The patient’s condition was assessed after four weeks, and the therapy regimen (at the same dose) was repeated. After more than two months of HON administration, an MRI scan was performed, during which tumor growth was not detected. No hepato-renal or hematologic toxicity was observed. However, an MRI scan 11 months after diagnosis indicated swelling at the tumor site. Therefore, the treatment included cisplatin with temozolomide, followed by bevacizumab in combination with etoposide, and carboplatin was introduced. Unfortunately, the patient died 19 months after diagnosis. This study highlighted that HON did not cause adverse effects, including hematologic toxicity [34]. Treatment of glioma includes the use of several drugs simultaneously to improve outcomes and reduce tumor recurrence. Therefore, micelles combining lauroyl-gemcitabine and HON (modified with hyaluronic acid) were developed to treat glioma. The best effect of lignan and lauroyl-gemcitabine was obtained when the ratio of the two substances was 1:1. The IC50 value for the developed micelles was 5.31 μM, while for the free drugs, it was 17.15 μM. The micelles could pass through the blood–brain barrier and accumulate in the tumor in mice. Induction of apoptosis and inhibition of tumor cell proliferation prolonged the survival time of mice [35]. Another interesting formulation designed to deliver HON to brain glioma is hydroxyapatite particles. The system released HON gradually, extending its duration of action compared to free HON. Studies in mice indicated a 40% reduction in tumor size and showed its therapeutic potential [36]. The discussed influence of HON on glioblastoma is presented in Figure 3.

## 4. Ovarian Cancer

Ovarian cancer is one of the most common causes of cancer-related deaths in women in developed nations. Women should be diagnosed and treated early for better chances of curing it to avoid the high rates of morbidity and mortality. In more than 7 of 10 women with ovarian cancer, the recurrence of cancer occurs after initial treatment [37]. HON has also been tested in the treatment of ovarian cancer. The main findings about the effect of the developed delivery system in this type of cancer are presented in Table 3.

**Figure 4:**
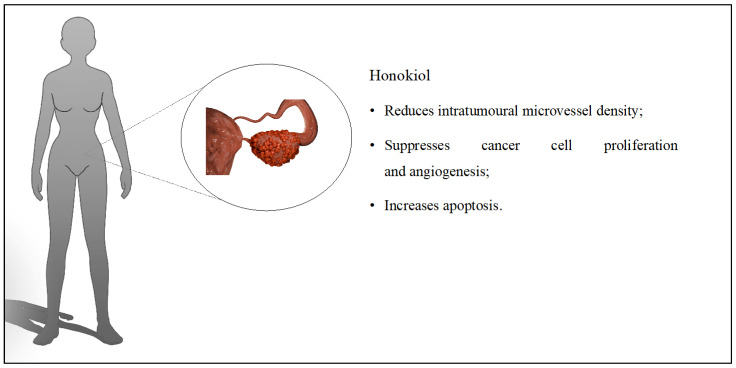
Key findings related to ovarian cancer.

Liposomes containing only HON were evaluated on cisplatin-sensitive and cisplatin-resistant ovarian cancer cells. In addition to in vitro studies, the antitumor effect of the system was determined on BALB/c mice. The used liposomes increased the number of apoptotic cells in A2780s (61.3%) and A2780cp (55.1%) cells. The designed system was administered to mice, and the results were compared with groups treated with empty liposomes, sodium chloride, and cisplatin. The tumor volume studied in A2780s cells was significantly reduced in the groups treated with liposomes loaded with HON and cisplatin with 222 mm^3^ and 112 mm^3^ values, respectively. However, there was no statistical difference between the groups. In contrast, the average tumor volume in A2780cp cells was equal to 408 mm^3^ for HON and 1607 mm^3^ for cisplatin. Mouse survival time was prolonged (in HON liposomes and cisplatin) compared to the control groups, and systemic toxicity was not registered. Moreover, one of the five mice experienced complete tumor regression [38]. HON-loaded liposomes were also combined with cisplatin. Using both compounds in one system had a more potent antitumor effect than when using them separately. Tumor weight and tumor volume were reduced by about 90%. Angiogenesis was reduced, and two out of five mice experienced tumor regression [39]. In another study, HON nanoparticles were prepared with doxorubicin. The physicochemical properties of the system and cytotoxicity on A2780s cells were evaluated. The average particle size was 230 nm, while the zeta potential was −5.502 mV. The nanoparticles containing doxorubicin to HON in the ratio of 1:3 had a stronger cytotoxic effect compared to compounds administered separately [40]. The discussed influence of HON on ovarian cancer is presented in Figure 4.

## 5. Lung Cancer

Lung cancer is the leading cause of cancer deaths around the world. Almost as many patients die of lung cancer every year than of prostate, breast, and colon cancer combined. The 5-year survival rate for lung cancer is about 15% in developed countries. Although there has been some improvement in survival during the past few decades, the effectiveness of treatment is still unsatisfactory [41]. The anticancer properties of HON were also assessed in different models of lung cancer. To improve the therapeutic effect of this compound, liposomes and micelles were developed. In Table 4, the summary of the studies on the biological effect of those formulations is presented.

Lung cancer cells are often preceded by EGFR dysfunction. In vitro studies have been conducted using cells at different stages of lung cancer development. Scientists proved that HON can inhibit proliferation by up to 93% after 72 h. This lignan is known to reduce the phosphorylation of EGFR, Akt, ERK, and STAT3 [49]. HON-loaded liposomes were administered to mice intranasally to investigate their antitumor potential. The performed measurements indicated that the developed system reduced the number of small (<0.5 mm) and large (0.5–1 mm) tumors. Similar to in vitro tests, in vivo experiments proved a reduction in EGFR, Akt, and STAT3 activation [42].

**Figure 5:**
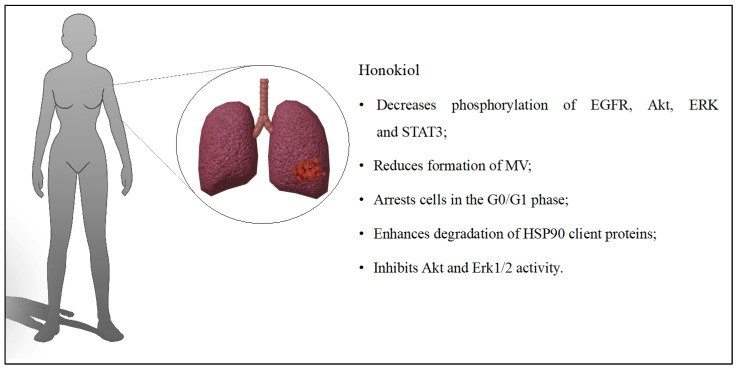
Key findings related to lung cancer.

In another study, liposomes were loaded with epirubicin and HON, and their surface was modified with octreotide to improve the targeting of cancer cells. The average size of the prepared particles was 108 nm. Encapsulation efficiency was above 90% for epirubicin and HON. The combination of epirubicin and HON on LTT cells significantly reduced their survival. Epirubicin alone also reduced survival, but the effect was smaller. Liposomes reduced the formation of VM channels. The developed system was detected in the mouse body for up to 24 h. The tumor volume after 12 days was 9.78 ± 3.19 for the prepared system, while it was 17.12 ± 5.79 for the epirubicin liposomes. The newly designed liposomes extended the survival time of mice to up to 48 days [43]. Jiang Qi-qi et al. [44] also evaluated the effect of liposomal HON combined with the anticancer drug cisplatin. In this case, only in vivo studies were conducted on mice with lung cancer. During the experiments, several aspects were evaluated, including tumor volume, development of angiogenesis, and apoptosis. The tumor volumes differed between groups: cisplatin (1264.27 mm^3^), liposomal HON (1028.72 mm^3^), or a combination of both substances (352.43 mm^3^). The number of vessels formed by the tumor was reduced in the study group. Similarly, liposomes increased the apoptosis of tumor cells, and toxicity was not observed in organs such as the liver, kidney, or lungs [44]. The simultaneous use of radiotherapy/chemotherapy with anti-angiogenic treatment could be an alternative to the traditional cancer therapy regimen. Cancer cells would be destroyed in the first stage, and in the second stage, they would be prevented from reappearing. The study proved that liposomal HON enhanced the effect of radiotherapy in in vitro and in vivo tests. Tumor size for the combined therapy decreased by 78% compared to the untreated tumor. The use of liposomes or radiotherapy reduced the tumor by 42%. It was determined that cells treated with both therapies were arrested in the G0/G1 phase. Liposomes with radiotherapy induced apoptosis better and reduced angiogenesis [45]. Liposomal HON was also evaluated on lung cancer cells resistant and sensitive to gefitinib. Lignan promoted the degradation of HSP90 client proteins (HCPs), thereby inhibiting Akt and Erk1/2 and directing cells to autophagy [46]. In another study, scientists selected four natural compounds with anticancer properties to assess them in liposomal formulations. They evaluated HON, betulinic acid, parthenolide, and ginsenoside Rh2, and their effects on lung cancer cells. The average size of the developed liposomes was 115.7 nm, and the encapsulation efficiency values ranged from 88.2% for ginsenoside Rh2 to 91.4% for HON. The four compounds combined in liposomes had a stronger cytotoxic effect on A549 cells than when the compounds were used separately. Tumor inhibition for liposomes was 60.7%, while for cisplatin, it was 65.1%. The system had antitumor activity in both in vitro and in vivo tests. However, when comparing the efficacy of the developed liposomes with cisplatin, the natural compounds exerted less of an antitumor effect [47]. Modified dequalinium micelles with paclitaxel combined with HON were evaluated in non-small cell lung cancer. The cytotoxic effect on LTT cells was examined in vitro. The highest effect was obtained for the developed micelles where the ratio of HON to paclitaxel was 1:1. The IC50 value was 0.16 µM ± 0.07 µM for the prepared micelles. In contrast, for the paclitaxel micelles, it was 1.89 µM ± 0.26 µM. The micelles destroyed VM channels and induced apoptosis. The percentage of apoptosis was 29.44% and 22.96% for the prepared system and micelles with paclitaxel, respectively. The in vivo studies confirmed four critical pieces of information about the designed system. The micelles accumulated in the tumor for up to 24 h, reduced tumor volume by 11.66 ± 3.47%, extended mouse survival time by up to 47 days, and did not induce systemic toxicity [48]. The discussed influence of HON on lung cancer is presented in Figure 5.

## 6. Liver Cancer

Liver cancer is the third leading cause of cancer deaths worldwide. The major etiologies for liver cancer are hepatitis B virus (HBV), hepatitis C virus (HCV), alcoholism, and non-alcoholic steatohepatitis (NASH) [50]. The role of the HON-loaded delivery system in liver anticancer therapy has recently been investigated. The main findings of those studies are described in Table 5.

Liver cancer can quickly spread to other organs. The EGF-EGFR pathway is involved in metastasis formation, activating PI3K/Akt, ERK, and JNK. HON can affect the previously mentioned pathways, but the effect may be negligible without an appropriate delivery system. Jianhong Yang et al. [51] evaluated HON-loaded liposomes in vitro and in vivo on liver cancer. The study was spread over several stages. First, the cytotoxicity of lignan on various cell lines was evaluated. Concentrations above 60 μM guaranteed cytotoxicity and cell apoptosis. Concentrations below 40 μM showed no such ability but had the potential to inhibit HepG2 migration and invasion. Studies on zebrafish confirmed that lignan inhibited the extravasation and survival of tumor cells. HON further inhibited the activity of pathways and enzymes such as ras-related C3 botulinum toxin substrate (Rac1), cell division control protein 42 homolog (Cdc42), and MMP-2 and -9. More importantly, HON inhibited EGFR and the PI3K/Akt, Erk, and JNK pathways in the conducted study. The in vivo study in mice also confirmed a 50% reduction in tumor volume after 24 days of treatment [51]. HON was also encapsulated into micelles using rebaudioside A. The system had very good physicochemical properties with an average particle size of 4.356 ± 0.142 nm. The lignan micelles arrested the cell cycle in the G0/G1 phase. The apoptosis rate was 57.2% and 32.7% for the developed system and free HON, respectively. Tumor inhibition was assessed during in vivo studies on BALB/c mice. The tumor inhibition rate was 30.72% in the free HON group and 72.77% in the micelles (at a dose of 100 mg/kg) [52].

**Figure 6:**
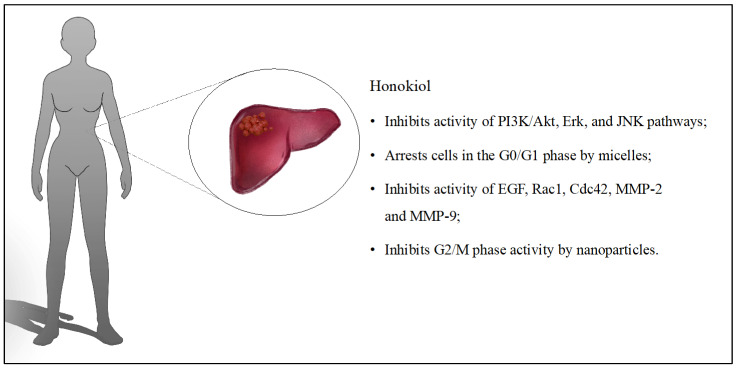
Key findings related to liver cancer.

In another study, scientists evaluated epigallocatechin-3-gallate (EGCG) functionalized chitin (CH) derivatives for the preparation of nanoparticles with HON. They were characterized by a spherical shape with an average size of 80 nm. In the first step, the toxicity of the system on healthy lung, kidney, and liver cells was evaluated. There was no increase in the number of apoptotic cells, and their viability remained high. HON nanoparticles caused a significant reduction in the survival of A549 (lung cancer) and HepG2 (liver cancer) cells after 24 h of incubation, at rates of 19% and 5%, respectively. The system inhibited activity at the G2/M stage, while free HON blocked the G1 phase. The good results in vitro encouraged in vivo testing in mice with subcutaneous HepG2 tumors. The animals were divided into a control group, with free HON at a dose of 40 mg/kg, HON-loaded nanoparticles at a dose of 20 mg/kg, and HON-loaded nanoparticles at a dose of 40 mg/kg. No inflammation or damage was present within the internal organs, nor were any other adverse effects registered in mice receiving nanoparticles. Free HON reduced tumor inhibition by approximately 30.15%, while nanoparticles reduced tumor inhibition by 83.55%, proving its high treatment potential [53]. The discussed influence of HON on liver cancer is presented in Figure 6.

## 7. Other Cancers

The anticancer therapeutic potential of HON is broad. Therefore, in the literature, single studies on various other types of cancer, such as colon cancer, tongue cancer, nasopharyngeal carcinoma, bone cancer, or melanoma, can also be found. The in vitro and in vivo findings of using different nanotechnology-based delivery systems of HON in those applications are presented in Table 6.

The potential combination of HON and cisplatin was evaluated in vitro and on mouse models of colon cancer. The authors expected the antitumor activity to increase when a combined therapy was applied. Indeed, apoptotic cells increased to 61.39% when combined with the lignan and the anticancer drug. This value was much higher than the expected value of 31.17%. The combination therapy reduced the tumor volume to 501.03 ± 263.80 mm^3^ in mice. Lignan, without the cisplatin, reduced the volume to 1813.87 ± 412.86 mm^3^, while cisplatin alone reduced it only to 2338.24 ± 531.29 mm^3^. No changes were detected in the studied organs (liver, heart, lungs, kidneys, and spleen) [54].

The potential anticancer effects of HON are not limited to cancers such as BC, lung cancer, and glioma. Applications would be much broader, including less commonly considered cancers. One study evaluated its effects on tongue cancer cells. Scientists developed titanium dioxide nanotubes. The proliferation, migration, and apoptosis of CAL-27 cells were determined. The nanotubes had a uniform structure on which HON was placed. The difference between cancer cell proliferation and migration levels was statistically significant between the free HON and nanotube groups. HON increased the expression of the Fax and Bas factors, thereby promoting apoptosis of cancer cells [55].

The effect of HON nanoparticles on nasopharyngeal cancer was investigated. Folate-modified poly(ε-caprolactone)-poly(ethyleneglycol)-poly(e-caprolactone) (PCEC) was used to obtain this system. The nanoparticles had good physicochemical properties, and the encapsulation efficiency was equal to 78.25%. The time required to release 90% of the loaded HON was 150 h, and the prepared system inhibited HNE-1 cells, with an IC50 value of 18.41 mg/mL. Modified nanoparticles with folate delayed tumor growth by 24 days. In comparison, free HON delayed tumor growth by eight days. The designed system extended the survival time of organisms with a median of 57.5 days compared to other groups, where the median was 28.5 days (saline group), 29.5 days (blank nanoparticles), 34 days (free HON), and 42.5 days (HON nanoparticles not modified with folate) [56].

Liposomal HON modified with hyaluronic acid-phospholipid conjugates was evaluated in osteosarcoma. The liposomes were characterized by a spherical shape and an average size of 146.2 ± 0.62 nm. The system was stable for seven days, and the encapsulation efficiency was 80.14 ± 0.32%. The system induced apoptosis and promoted the arrest of the cell cycle in the G1 phase. It also revealed good penetration into tumor cells during in vivo studies. The last group showed the best antitumor effects by comparing tested groups treated with PBS, free HON, liposomes with HON, and modified liposomes with HON. Moreover, the tumor weight decreased significantly without causing weight loss or organ damage [57].

Gong ChangYang et al. [58] developed HON micelles on a thermosensitive hydrogel. The system exhibited very good physicochemical properties. Empty micelles and unloaded hydrogel were biocompatible and had no cell cytotoxicity. Moreover, HON micelles on the hydrogel exert a slow release of the lignan with a release time of up to two weeks. HON inhibited the growth of B16 melanoma cells in dose-dependent in vitro experiments, showing its high potential for further studies [58].

## 8. Other Cancer-Related Effects

The anticancer effect of *Magnolia officinalis*-derived lignans, especially HON is well-studied in different cancer models; however, their effect in reducing cisplatin toxicity, malignant pleural effusions, and angiogenesis or lymphangiogenesis is less obvious.

Cisplatin is an anticancer drug used as monotherapy or combined with other compounds. The mechanism of action involves the formation of cross-links within DNA. Although it has efficacy in cancer, cisplatin is characterized by nephrotoxic and ototoxic effects. Fewer side effects are associated with its replacement with oxaliplatin or carboplatin. However, they may show less efficacy against cancer. Cisplatin’s nephrotoxicity is related to its accumulation in the renal tubules, among other factors. Liu Hung-Ting et al. [59] experimented using a HON nanosuspension to reduce nephrotoxicity after cisplatin treatment. The study consisted of several phases. In the first stage, kidney damage was induced in mice using cisplatin. After determining the lowest effective dose of lignan, the researchers performed an in vivo study. They used a nanosuspension at a selected dose and compared the results with control groups. The lowest effective dose of HON nanosuspension was 5 mg/kg. The prepared system was evaluated for its effects on body weight, urine osmolarity, serum blood urea nitrogen, creatinine, and serum indoxyl sulfate. The nanosuspension improved creatinine, body weight, and serum blood urea nitrogen parameters. HON also reduced the number of inflammatory cells and fibrosis in the kidneys. The group treated only with cisplatin exhibited a reduced expression of cytochrome c, which is responsible for redox reactions in the mitochondria. Cytochrome c activity was partially recovered using lignan nanosuspension. In addition, the system reduced cell apoptosis by acting on caspase 3. The author concluded that HON nanosuspension protected kidneys after cisplatin treatment [59].

Malignant pleural effusions can occur in patients with advanced stages of cancer. This condition reduces quality of life and increases patient mortality. Scientists evaluated the effect of HON nanoparticles in the hydrogel on malignant pleural effusions. HON nanoparticles in the hydrogel were released much more slowly than HON in nanoparticles. After 72 h, the lignan release from the developed formulation was 33% and 10% for nanoparticles and hydrogel, respectively. Prolonged release (from the hydrogel) would provide less toxicity to healthy tissues and prolong action at the tumor site. The properties of the new system were determined on mice inoculated with LLC cells. The condition of the organisms was assessed using CT scans. Nanoparticles better reduced the number of tumor foci and pleural effusions in the hydrogel compared to other groups. In addition, the treatment increased apoptosis and survival time to an average of 25 days. In the other groups, survival times were 16 and 18 days [60].

Metastasizing cancer complicates treatment and worsens patient outcomes. Lymphangiogenesis may contribute to tumor cell transport and lymph node metastasis. Increased lymphangiogenesis is induced by VEGFR-3, VEGF-C, and/or VEGF-D. HON, which acts on these receptors, may solve this problem. The effects of liposomal HON were determined in in vitro studies on HUVEC and HLEC cell lines.

In contrast, in vivo studies were performed on C57BL/6 mice injected with Lewis lung cancer cells overexpressing VEGF-D. The proliferation and survival of VEGF-D-induced cells were reduced during in vitro studies. Cell neogenesis was inhibited in the presence of 50 μmol/L HON. Changes in the characteristics of apoptosis were noted in the studied cells. Western blot analysis showed decreased phosphorylation of Akt and p44/42 mitogen-activated protein kinase, which decreased the expression of VEGFR-2 and VEGFR-3. The inhibition of angiogenesis or lymphangiogenesis was assessed by tube formation assay. Tests on HLEC cells with HON indicated a reduction in tube formation. In comparison, the absence of lignan resulted in the forming of a rich tube network. Tumors grew faster in the placebo-treated group than liposomal HON (25 mg/kg/day and 50 mg/kg/day dose). In addition, all doses of HON (12.5–50 mg/kg/day) increased the survival time of the mice. On day 46 of treatment, 80% of them (taking the 50 mg/kg/day dose) were alive. Only one mouse out of ten, treated with lignan at 50 mg/kg/day, developed lymph node metastases. In comparison, 7 out of 10 (free liposomes) and 8 out of 10 (saline-treated) test organisms developed metastases. HON has been shown to be beneficial in inhibiting lymphangiogenesis [61].

## 9. Anticancer Effect of Magnolol—Positional Isomer of Honokiol

Magnolol (MAG) and HON are positional isomers; however, this subtle difference in their chemical structure affects their activity and mechanism of action. Although MAG is not as popular as HON among scientists studying the anticancer effects of compounds of natural origin, several studies indicate the potential of this compound in this field.

Mona Elhabak et al. [62] evaluated the anticancer effects of MAG on BC. During this study, the effect of the MAG loaded into nanoparticles was evaluated on BC cells. Apart from lignan, the system contained trastuzumab, gold nanoparticles, PLGA, and TPGS. A spherical and uniform shape characterized the prepared nanoparticles. The system ensured a slow release of the compound. Within 48 h, 50.24 ± 2.41% of MAG was released from the nanoparticles, whereas the release of free MAG was 97%. MTT assay was performed to assess the viability of MCF-7 cells. Developed MAG-loaded nanoparticles with trastuzumab showed the greatest reduction in cell viability. MAG encapsulated in nanoparticles inhibited cell activity better than pure lignan. The IC50 value of lignan decreased from 2.92 to 1.81 when the compound was incorporated into the nanoparticles. In conclusion, the prepared system could be an alternative for treating BC, exploiting the anticancer potential of MAG [62].

Yanzhi Wang et al. [63] studied the effect of nanoparticles on TNBC cells. The polymeric nanoparticles consisted of cholesterol biguanide conjugate hydrochloride (CBH), poly(ethylene glycol)-poly(lactic-co-glycolic acid) (mPEG-PLGA), aminoethyl anisamide-poly(ethylene glycol)-poly(lactic-co-glycolic acid) (AEAA-PEG-PLGA), and MAG. CBH was used as the drug and carrier for the MAG, while AEAA-PEG-PLGA targeted the nanoparticles to cancer cells. The in vitro studies were performed on 4T1 cells and in vivo on female BALB/c mice with developed tumors. Among others, the analyses determined tumor or normal cell apoptosis, inhibition of tumor growth, and levels of cell regulatory factors and toxicity of the system on mouse organs were performed. AEAA-PEG-PLGA provided enhanced tumor cell targeting. The highest distribution to the tumor cells was achieved when the ratio of AEAA-PEG-PLGA to mPEG-PLGA was 4:1. The mean diameter of the resulting nanoparticles was 145.4 nm, while the zeta potential was 14. The physicochemical properties of nanoparticles remained unchanged for a further six months. In combination with CBH, MAG showed a better cytotoxic effect on 4T1 cells and had a higher overall apoptosis rate (approximately 70%). The prepared system did not cause systemic toxicity in mice. At the same time, a significant reduction in tumor volume was registered. The volume inhibition rate was ultimately 77.0 ± 13.8%. Western blot analysis determined cellular protein activity, showing that mangolol with CBH upregulated the expression of tumor protein p53, phospho-AKT, and AMP-activated protein kinase. The antitumor effect of the developed nanoparticles was confirmed in vitro and in vivo and could provide a new treatment option for TNBC [63].

## 10. Conclusions and Future Perspective

The number of cancer diagnoses increases in the population each year. Cancer can be detected early through routine screening of patients. Intensive treatment after early diagnosis offers the chance of complete remission of the cancer. However, there are still treatments that possess limitations or are ineffective for some cancers, resulting in a low chance of a cure. Currently, many studies are focusing on the application of natural compounds in the development of anticancer treatments.

According to the presented literature, HON exhibits broad anticancer effects. However, its mechanism of action is not yet fully understood. There are several pathways recognized as being involved in its anticancer effect. Nevertheless, further studies are necessary to determine the exact mechanism and site of action, including the investigation of other active lignans, especially MAG which, although a positional isomer of HON, may present a different strength of action or even act through other biochemical pathways. In addition, the activity of the metabolites of MAG and HON would be worth assessing, as they may also affect cells. Such studies would further help to determine the safety of the application of lignans in patient therapy.

The interesting approach undertaken by several authors combined classical anticancer drugs with HON to improve the efficiency of applied therapy. Combining two compounds may complement each other, providing better effects and/or potentially allowing the use of lower doses of anticancer drugs, which may reduce some side effects.

The poor water solubility of HON requires advanced delivery systems to take full advantage of its capabilities. So far, HON has been introduced into liposomes, micelles, nanocapsules, polymeric nanoparticles, nanosuspensions/hydrogels, microparticles, and nanotubes for its utilization as an anticancer medication. The systems reduce premature metabolism and increase the duration of action at the appropriate site. Further studies should include evaluating individual systems in a given tumor to select the best and most promising system.

## Figures and Tables

**Table 1:** The effect of honokiol (HON) delivery systems on breast cancer (BC).

Main Compounds	Carrier	Aim of the Study	Disease; In Vitro/In Vivo	Conclusion
HON + polysialic acid [11]	Liposomes	Preparation of HON liposomes modified with polysialic acid and evaluation of their anticancer activity.	BC; in vitro (4T1) and in vivo (BALB/c mice)	The IC50 value was 4.84 μg/mL; liposomes reduced the concentration of HON required to achieve the same antitumor effect. The tumor volume in mice was reduced by 52% with liposomes. This effect was probably due to the accumulation of HON and the presence of polysialic acid.
HON + hyaluronic acid [12]	Liposomes	Preparation of modified liposomes with HON and preliminary evaluation of their anticancer activity in vitro and in vivo studies.	BC; in vitro (4T1) and in vivo (BALB/c mice)	Cytotoxicity was greater with hyaluronic acid-modified micelles. The liposomes had greater accumulation in tumor cells and 59.5% inhibition of tumor growth. There was no systemic toxicity manifested by weight loss in the test subjects.
HON + adriamycin [13]	Liposomes	Determination of the anticancer effect of adriamycin with HON on cancer cells in vitro and in vivo studies.	BC; in vitro (4T1) and in vivo (BALB/c mice)	HON reduced the IC50 value for adriamycin by up to 15 times in vitro. The combined compounds induced apoptosis of cancer cells. The survival time of the test organisms was extended to 51 days (compared to 43 days for adriamycin).
HON + daunorubicin + hyaluronic acid [14]	Liposomes	Designing modified liposomes with HON to determine their effect on BC.	BC; in vitro (MCF-7 and MDA-MB-435S) and in vivo (BALB/c mice)	In vitro as well as in vivo studies confirmed the inhibitory effect of liposomes on cancer cells. Liposomes had higher cytotoxicity and provided prolonged release of substances, showing limited systemic toxicity in mice.
HON + paclitaxel [15]	Micelles	Designing micelles to improve anticancer activity of selected compounds.	BC; in vitro (4T1 and HEK293) and in vivo (BALB/c mice)	HON and paclitaxel in micelles had high cytotoxicity against cancer cells compared to other tested samples. The prepared system had also a higher percentage of apoptosis processes (34.02 ± 0.05%), inhibited angiogenesis, and suppressed the proliferation of tumor cells more effectively.
HON + paclitaxel [16]	Micelles	Use of micelles with HON and paclitaxel to prevent metastasis of BC and suppress multidrug resistance.	BC; in vitro (MCF-7/ADR and MDA-MB-231) and in vivo (BALB/c mice)	The micelles inhibited cell invasion regardless of the concentration of compounds in the system. Cell migration dropped to 66.6% with simultaneous paclitaxel and HON in the micelles. Bioluminescence imaging system proved that the administered system inhibited metastasis formation.
HON + doxorubicin [17]	Micelles	Prevention of metastasis during BC through the use of HON combined with the anticancer drug doxorubicin.	BC; in vitro (MDA-MB-231) and in vivo (BALB/c mice)	Migration and invasion of cancer cells were suppressed by created micelles. Adverse effects of doxorubicin were limited due to the reduction in premature drug release.
HON [18]	Micelles	Determination of antitumor effect of HON on triple-negative BC cells.	Triple-negative BC; in vitro (MDA-MB-231, MDA-MB-453, and MDA-MB-468) and in vivo (BALB/c mice)	Conducted studies demonstrated an increase in Cmax and AUC following oral administration of micelle-encapsulated HON. In addition, in vivo studies showed a reduction in tumor weight. In some individuals, complete recovery was observed.
HON + celecoxib [19]	Micelle solution	Developing and evaluating a mixed micelle solution to treat BC.	BC; in vitro (4T1) and in vivo (BALB/c mice)	The use of micelles resulted in a 45.71% apoptosis of cancer cells. In addition, there was a reduction in the expression of tumor growth biomarkers and a reduction in the number of collagen fibers in tumor tissue.
HON [20]	Nanocapsules	Systemic use of HON in the treatment of BC.	BC; in vitro (MCF-7 and EAC) and in vivo (BALB/c mice)	Conducted tests showed that using HON-loaded nanocapsules resulted in an 80% inhibition of tumor growth and an 85% reduction in tumor weight. As a result, the prepared system had very good results both in the in vitro and in the in vivo studies.
HON + zein + polysialic acid [21]	Polymeric nanoparticles	Prevention of BC growth and inhibition of metastasis by using HON encapsulated in nanoparticles.	BC; in vitro (4T1) and in vivo (BALB/c mice)	Polysialic acid nanoparticles inhibited tumor growth more strongly, increasing the penetration of the system into cells. Tumor growth was inhibited by 52%. The applied system reduced metastasis to other organs.
HON + zein + hyaluronic acid [22]	Polymeric nanoparticles	Development of core–shell nanoparticles to improve cellular uptake and distribution to enhance the antitumor effects of HON and inhibit metastasis.	BC; in vitro (4T1) and in vivo (BALB/c mice)	Nanoparticles were more effective in inhibiting tumor growth (77%) compared to free HON (25.8%). Mitochondrial pathway was involved in the apoptosis process. The encapsulation of HON in nanoparticles increased the efficacy of the compound in vivo, where antitumor activity was observed even at a dose of 15 mg/kg.
HON + polydopamine + folic acid [23]	Polymeric nanoparticles	Preparation of nanoparticles containing HON, polydopamine, and folic acid for the targeted treatment of cancer.	BC; in vitro (4T1) and in vivo (BALB/c mice)	The IC50 value was 2.25 after 48 h of incubation for the prepared system with polydopamine and folic acid. The particles were mainly captured by tumor cells. In vivo studies indicated a tumor growth inhibition of 79%.
HON + camptothecin + chitosan + folic acid [24]	Polymeric nanoparticles	Determination of the effect of HON nanoparticles on BC cells.	BC; in vitro (MCF-7)	In vitro studies confirmed the inhibition of cancer cell growth by the nanoparticles.
HON [25]	Polymeric nanoparticles	Increasing the use of poorly soluble HON in the treatment of BC and prevention of lung metastasis.	BC and lung metastases; in vitro (4T1) and in vivo (BALB/c mice)	The percentage of apoptotic cells was 64% with the designed nanoparticles. The same parameter was only 5% with free HON. The nanoparticles also reduced tumor volume and tumor growth at a dose of 15 mg/kg HON.
HON + amphiphilic codendrimer + oligoethylene glycol [26]	Polymeric nanoparticles	Design of dendrimer-based nanoparticles for precise delivery of HON.	BC; in vitro (4T1) and in vivo (BALB/c mice)	In vitro, after 48 h, the IC50 value for the prepared system was 2.2 μg/mL. The same parameter for free HON was 5.7 μg/mL. The system had a higher cytotoxicity on cancer cells, probably due to the greater transport of the compound into the cells. In vivo studies showed that HON nanoparticles (at a dose of 40 mg/kg) inhibited tumor growth by 72% compared to a saline-treated control group.
HON + paclitaxel [27]	Nanosuspensions/hydrogel	Combined anticancer activity of HON hydrogel suspension and paclitaxel for the treatment of BC.	BC; in vitro (4T1) and in vivo (BALB/c mice)	The combination of HON and paclitaxel enhanced the cytotoxic effect of both compounds. The apoptosis rate was more than 22% for HON in hydrogel, and when administered with paclitaxel, the rate increased to 65%. In vivo studies showed that paclitaxel with HON in hydrogel had the strongest antitumor effect at a dose of 40 mg/kg.

**Table 2:** The effect of honokiol (HON) delivery systems on glioma.

Main Compounds	Carrier	Aim of the Study	Disease; In Vitro/In Vivo	Conclusion
HON + disulfiram + copper + binding peptide (^D^CDX) [30]	Liposomes	^D^CDX peptide-modified HON liposomes for the treatment of glioblastoma.	Glioblastoma; in vitro (U87, C6, and BEND3) and in vivo (BALB/c mice)	The prepared liposomes showed a higher antitumor effect compared to free drugs. Liposomes induced autophagy and apoptosis by inhibiting Akt/mTOR phosphorylation. The average survival time of the tested mice was 27 days, of which 30% were still alive at the end of the study.
HON [31]	Liposomes	Determining the mechanism of action of liposomes in the treatment of glioblastoma.	Glioblastoma; in vitro (U87, LN229, RAW264.7, and BV2) and in vivo (ICR mice)	Studies have shown that HON liposomes act on tumor-associated macrophages. The antitumor lignan modulates their activity by increasing M1 polarization and inhibiting M2 polarization.
HON + daunorubicin + lactoferrin [32]	Liposomes	Designing liposomes to cross the blood–brain barrier and use them in brain glioma.	Glioblastoma; in vitro (C6 and BMVECs) and in vivo (ICR mice)	In vitro studies showed that the survival rate of tumor cells was 3% after use of the prepared system. The liposomes were better at inhibiting the formation of VM channels. The used liposomes prolonged the survival time of the mice in the experiment. This ranged from 23 to 51 days.
HON + doxorubicin [33]	Micelles	Utilizing the antitumor effects of HON and doxyrubicin for the treatment of malignant glioma.	Malignant gliomas; in vitro (C6) and in vivo (zebrafish model embryos and BALB/c mice)	HON in combination with doxorubicin more effectively inhibited cell proliferation and angiogenesis and induced apoptosis. Moreover, both substances showed synergistic effects when used together.
HON [34]	Liposomes	Evaluation of safety and efficacy of HON-loaded liposomes in a patient with glioblastoma.	Glioblastoma; case study	The patient did not experience any serious side effects. There were no elevated liver enzymes or blood counts. The HON administered supported the patient’s treatment with anticancer drugs.
HON + lauroyl-gemcitabine + hyaluronic acid [35]	Micelles	Combining HON with gemcitabine to exploit their properties in the treatment of glioblastoma multiforme.	Glioblastoma multiforme; in vitro (U87, B16F10, and HK2) and in vivo (SD rats and BALB/c mice)	The compounds showed a synergistic effect by inhibiting glioma progression. The micelles produced led to uptake by the tumor cells, greater accumulation of the drug at the site of action, and prolonged life of the mice due to greater induction of apoptosis and inhibition of cell proliferation.
HON + hydroxyapatite + stearic acid [36]	Microparticles	Use of hydroxyapatite to deliver HON to target cancer cells.	Glioblastoma; in vitro (ALTS1C1) and in vivo (C57BL/6 mice)	In vitro, as well as in vivo studies, indicated that the designed system provided controlled release of HON. Compound promoted tumor cell apoptosis and significantly reduced tumor size compared to control groups.

**Table 3:** The effect of honokiol (HON) delivery systems on ovarian cancer.

Main Compounds	Carrier	Aim of the Study	Disease; In Vitro/In Vivo	Conclusion
HON [38]	Liposomes	Evaluation of the effect of HON on ovarian cancer cells.	Ovarian cancer; in vitro (A2780s and A2780cp) and in vivo (BALB/c mice)	Liposomal HON promoted apoptosis in cell lines (A2780s and A2780cp) in vitro. The system reduced tumor volume in mice during in vivo studies.
HON + cisplatin [39]	Liposomes	Use of PEG liposomal HON with cisplatin for the treatment of ovarian cancer.	Ovarian cancer; in vitro (SKOV3) and in vivo (BALB/c mice)	The prepared liposomes induced apoptosis in vitro at a dose of 1 μg/mL. The mean tumor volume was 403 mm^3^ after application of the liposomes. Compared to the control group, tumor volumes were 90% lower.
HON + doxorubicin [40]	Polymeric nanoparticles	Determination of the anticancer potential of manufactured nanoparticles on ovarian cancer cells.	Ovarian cancer; in vitro (A2780s)	Compared to free doxorubicin and HON at the same doses, nanoparticles containing both compounds were more effective at inhibiting tumor cell proliferation.

**Table 4:** The effect of honokiol (HON) delivery systems on lung cancer.

Main Compounds	Carrier	Aim of the Study	Disease; In Vitro/In Vivo	Conclusion
HON [42]	Liposomes	Evaluation of HON-loaded liposomes against lung cancer.	Lung cancer; in vitro (BEAS-2B, 1179, 1198, and 1170) and in vivo (A/J mice)	HON inhibited phospho-extracellular signal-regulated kinases (ERK), Akt, and STAT3, while it promoted CD2 or CD4. Decrease in epidermal growth factor receptor (EGFR) phosphorylation was registered 6 h after administration. The compound showed an antitumor effect, by reducing proliferation (93%) and inducing apoptosis (61%). Similar results were obtained during in vivo studies in A/J mice. The test organisms showed a reduction in the size of lung tumors regardless of their size. Liposomes reduced EGFR, Akt, and STAT3 activity. Concomitant use of HON and erlotinib showed a stronger antitumor effect compared to the compounds separately.
HON + epirubicin + octreotide [43]	Liposomes	Preparation and evaluation of multifunctional liposomes for the treatment of non-small cell lung cancer.	Non-small cell lung cancer; in vitro (LLT) and in vivo (C57BL/6 mice)	The study found that multifunctional epirubicin liposomes exhibited the strongest cytotoxicity compared to other groups. The system provided increased penetration into tumor cells. The liposomes reduced tumor volume in test mice and prolonged survival to 48 days.
HON + cisplatin [44]	Liposomes	Determination of the antitumor activity of HON and evaluation of cisplatin potentiation by lignan.	Lung cancer; in vivo (nude mice inoculated with A549 cells)	The concentration of HON was above 30 μg/mL 24 h after liposome administration. In contrast, free HON concentrations were below 5 μg/mL after 12 h. The combination of HON and ciplastin inhibited tumor growth to a greater extent than the control groups.
HON [45]	Liposomes	Evaluation of radiotherapy enhancement by HON encapsulated in liposomes.	Lung cancer; in vitro (SPC-A1, A549, and LL/2) and in vivo (C57BL/6 mice)	Use of liposomes and radiotherapy together reduced tumor volume by 78% compared to untreated tumors. The therapies used separately resulted in a 42% reduction in tumor volume. Tumor growth was also delayed by more than 8 days. The combined therapies also reduced angiogenesis, and the average number of blood vessels was 16.2.
HON [46]	Liposomes	Liposomal HON for the treatment of non-small cell lung cancer of both gefitinib-sensitive and gefitinib-resistant cells.	Non-small cell lung cancer; in vitro (H1975, HCC827, H460, SPC-A1, H441, H1650, H226, H522, and H1993)	HON caused the degradation of HSP90 client proteins. This occurred via the lysosomal pathway. In a further step, misfolded proteins appeared, leading to ER stress and cell autophagy.
HON + betulinic acid + parthenolide + ginsenoside Rh2 [47]	Liposomes	Harnessing the anticancer effects of natural compounds in lung cancer.	Lung cancer; in vivo (A549) and in vivo (nude mice injected with A549 cells)	Liposomes inhibited the cell cycle in the G2/M phase, as the reference drug cisplatin. The rate of apoptosis was half that of cisplatin. The difference between the two groups was 4.40% for tumor growth in in vivo studies.
HON + paclitaxel + dequalinium [48]	Micelles	Development of a delivery system targeting VM cannel inhibition and tumor cell suppression.	Non-small cell lung cancer; in vitro (LLT) and in vivo (C57BL/6 mice)	Prepared micelles decreased regulation of FAK, phosphatidylinositol-3-kinase (PI3K), MMP-2, and MMP-9. Micelles had the strongest cytotoxic effects, reduced the formation of VM channels, and induced apoptosis of LLT cells. During in vivo test, delivery system increased the accumulation of chemotherapeutic drugs.

**Table 5:** The effect of honokiol (HON) delivery systems on liver cancer.

Main Compounds	Carrier	Aim of the Study	Disease; In Vitro/In Vivo	Conclusion
HON [51]	Liposomes	Using HON’s inhibitory effect on EGFR to suppress metastasis in hepatocellular carcinoma.	Hepatocellular carcinoma; in vitro (HepG2) and in vivo (zebrafish embryos and nude mice injected with HepG2/B16F10 cells)	HON inhibited and led to EGFR degradation and had inhibitory effects on the PI3K/Akt, Erk, c-Jun N-terminal kinases (JNK) pathways. A dose of 20 mg/kg reduced metastasis formation in the lungs of the mice tested. In addition, HON caused inhibition of tumor growth by 50% compared to control groups.
HON + rebaudioside A [52]	Micelles	Evaluation of prepared HON micelles with rebaudioside A on cancer cells.	Hepatocellular carcinoma; in vitro (HuH-7 and H22) and in vivo (BALB/c mice)	The survival rate of cancer cells treated with micelles containing HON was 15.69% during in vitro study. HON micelles inhibited the G0/G1 cell cycle by almost 82%. Depending on the dose administered, in vivo studies showed an inhibition of tumor growth of between 43% and 72%.
HON + epigallocatechin-3-gallate + chitin [53]	Polymeric nanoparticles	Determining the properties of HON nanoparticles on liver cancer.	Liver and lung cancer; in vitro (HepG2 and A549) and in vivo (BALB/c mice—liver cancer)	Nanoparticles inhibited tumor growth by almost 84%, compared to free HON, where the value was 30%. The nanoparticles had low systemic toxicity and stopped the cycle of tumor cells in the G2/M phase.

**Table 6:** The effect of honokiol (HON) delivery systems on other cancers.

Main Compounds	Carrier	Aim of the Study	Disease; In Vitro/In Vivo	Conclusion
HON + cisplatin [54]	Liposomes	Enhancement of the antitumor effect of cisplatin on colon cancer cells by HON.	Colon cancer; in vitro (CT26) and in vivo (BALB/c mice)	The combination of cisplatin and HON promoted tumor cell apoptosis (just under 62%), compared to 21% with cisplatin and 20% with HON. Analysis at 32 days showed that tumor volume was 501 mm^3^, demonstrating greater suppression with the drug combination.
HON + titanium dioxide [55]	Nanotubes	Evaluation of the anticancer effect of HON in nanotubes on tongue cancer cells.	Tongue cancer; in vitro (CAL-27)	The prepared nanotubes with HON inhibited proliferation and promoted apoptosis of cancer cells due to increased expression of apoptotic proteins.
HON [56]	Polymeric nanoparticles	Development of a novel drug delivery with HON for the treatment of nasopharyngeal carcinoma.	Nasopharyngeal carcinoma; in vitro (HNE-1) and in vivo (BALB/c mice)	The nanoparticles inhibited HNE-1 cell growth more effectively than free HON and extended the life span of the mice in the study to almost 56 days.
HON + hyaluronic acid-phospholipid conjugates [57]	Liposomes	Determining the effect of HON liposomes in bone cancer in in vitro and in vivo studies.	Osteosarcoma; in vitro (143B) and in vivo (nude mice injected with 143B cells)	Liposomes enhanced the anticancer effects of HON. Scientists observed increased apoptosis and inhibition of cancer cell proliferation.
HON [58]	Micelles/hydrogel	Development of HON micelles in a thermosensitive gel to increase the solubility of the compound and enhance its potential use in medicine.	Melanoma; in vitro (B16)	The prepared system inhibited cell growth dose-dependently and provided prolonged HON release.
